# SBA-15 Mesoporous Silica Modified with Gallic Acid and Evaluation of Its Cytotoxic Activity

**DOI:** 10.1371/journal.pone.0132541

**Published:** 2015-07-07

**Authors:** Dawid Lewandowski, Piotr Ruszkowski, Anita Pińska, Grzegorz Schroeder, Joanna Kurczewska

**Affiliations:** 1 Faculty of Chemistry, Adam Mickiewicz University, Poznan, Poland; 2 Faculty of Pharmacy, Poznan University of Medical Sciences, Poznan, Poland; University of Quebect at Trois-Rivieres, CANADA

## Abstract

Gallic acid has been covalently conjugated to SBA-15 mesoporous silica surface through different linkers. Cytotoxic activity of the hybrid organic-inorganic systems against HeLa and KB cell lines has been analyzed. Up to 67% of HeLa or KB tumor cells growth inhibition has been achieved at low silica concentration used (10 μg mL^-1^).

## Introduction

Mesoporous silicas discovered in the early 1990s have found numerous applications in science and industry because of their versatility, high surface area, thermal resistance and ease of surface functionalization. The last feature can be used for the enhancement of adsorption properties [1,2], ion exchange [3], catalytic properties [4,5] or cargo delivery [6,7]. SBA-15 mesoporous silica, with pore diameter ranging between 4.0 and 30.0 nm [8,9] and hexagonal pore order, can be utilized in each of these fields.

The use of SBA-15 mesoporous silica in the preparation of controlled drug release systems is well known [10] and anticancer drugs, besides the anti-inflammatory drugs, have been most intensively delivered in such systems. Most of these systems rely on the adsorption properties of anticancer drugs and gate-like structures located at the pore entrances [11] or on surface modifications [12] affecting the adsorption process. Covalent conjugation of the drug to the silica surface has been seldom reported [13]. The probable reason is that physically adsorbed anticancer drugs need only to be transported, using mesoporous silica carriers, to the vicinity of target tumor cells and protected from premature release by different stimuli-sensitive moieties. Covalently bound drugs require endocytosis of the silica particles by the tumor cells as already been reported in literature[14]. The addition of covalently conjugated folic acid enhances the particles uptake[14,15].

Polyphenolic compounds occur commonly in nature and play an important role in natural processes and ecology of plants. Less frequently they can also be found in animals. Polyphenols have been proved to show anticancer activity via many mechanisms of action [16]. Gallic acid is a triphenol derivative of benzoic acid and has been studied intensively towards anticancer properties either solely [17,18] as well as a part of more sophisticated systems, like magnetic nanoparticles [19,20]. The mechanisms of anticancer behavior of polyphenols have not been definitely solved yet. Some authors have suggested mobilization of chromatin-bound copper and prooxidation leading to cell death [21], while others point out cell stress damaging cellular integrity and functionality [22] or high structure dependence on polyphenol compound activity [23].

To the best of our knowledge, gallic acid in any form has not been successfully grafted onto the mesoporous silica nanoparticles surface. The aim of this study was to covalently conjugate gallic acid to SBA-15 mesoporous silica and analyze cytotoxic activity of these complex systems.

## Materials and Methods

### Materials

Gallic acid (GA, ≥98.0%) and 3-(2-aminoethylamino)propyltrimethoxysilane (AMETAM, ≥98.0%) were purchased from Fluka, polyethylenimine (PEI, M_w_~2000, 50% wt. solution in water), (3-aminopropyl)trimethoxysilane (APTMS, 97%), (3-chloropropyl)trimethoxysilane (CPTMS, 97+%), folic acid (FA, ≥97%), diisopropylcarbodiimide (DIC, ≥98.0%), N,N-diisopropylethylamine (DIPEA, ≥99.0%) and all solvents used in the study were purchased from Sigma-Aldrich and used without further purification. SBA-15 mesoporous silica (8–11 nm pore diameter, 600 m^2^ g^-1^ surface area and 1–2 μm particle size) was purchased from ACS Material.

### Preparation of gallic acid derivatives

In the first step gallic acid was converted to its tri-O-acetyl derivative using the procedure adapted from Ye et al. [24]. A portion of 2.90 g of gallic acid was placed in a flask to which 10.0 ml (~6.2 eq) of acetic anhydride was added. The mixture was stirred while 15 μl of concentrated sulfuric acid was added. The temperature rose up to about 60°C and the mixture became a clear solution. It was allowed to cool to the room temperature and 60 ml of water was added. After stirring for 2 h, the white precipitate was filtered off, washed thoroughly with water and dried under reduced pressure. The amount of 4.29 g of acetyl-protected gallic acid was obtained, which is 86% of theoretical yield. The purity was confirmed by melting temperature determination (Mel-Temp melting point apparatus), electrospray mass spectrometry (Micromass ZQ spectrometer, Waters) and IR spectroscopy (FT-IR spectrometer IFS 66/s, Bruker).

**Fig 1:**
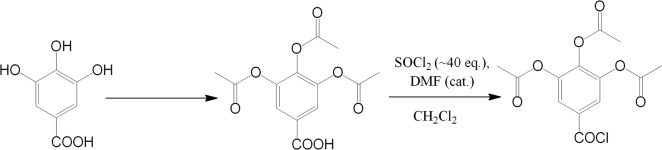
Modifications of gallic acid enabling its introduction onto the silica surface.

Acetylated gallic acid was converted into its acyl chloride each time before the immobilization on the surface. The procedure was as follows: tri-O-acetylgallic acid was dissolved in a small amount (a few ml) of dichloromethane and then large excess of thionyl chloride (~40 eq) was added, followed by catalytic amounts of dimethylformamide. The mixture was refluxed for 2 h and then volatiles were evacuated *in vacuo* (**Fig 1**). Residual amounts of unreacted thionyl chloride were removed by co-evaporation with toluene. Remaining solid was dissolved in toluene and all toluene-insoluble impurities were eliminated by filtration.The final product was obtained as white, crystalline solid with almost (>95%) quantitative yield.

### Immobilization of gallic acid derivatives on the SBA-15 mesoporous silica surface

All gallic acid derivatives were immobilized on the silica surface through amine containing groups: APTMS, AMETAM and PEI.

The preparation of SBA-15 mesoporous silica covered with APTMS and GA (named SBA-15+APTMS+GA) was conducted as follows: 0.300 g of SBA-15 mesoporous silica was suspended in 10 ml of toluene and an excess (1.000 g) of APTMS was added. The mixture was refluxed for 3 h and then stirred overnight at room temperature, filtered off, washed with toluene and dried. The amount of 0.362 g of APTMS-modified SBA-15 silica was obtained. In the second step 0.360 g of SBA-15+APTMS was suspended in 5 ml of THF and a small excess of DIPEA was added, followed by the addition of 0.425 g of tri-O-acetylgalloyl chloride dissolved in 5 ml of THF (that is 2–3 times higher with respect to the amount of NH_2_ groups on the surface). The mixture was stirred overnight at room temperature and then filtered off. The solid was washed carefully with THF, methanol and water to remove all soluble impurities, and dried. The amount of 0.427 g of SBA-15+APTMS+GA as a white solid was obtained and analyzed using elemental analysis and IR spectroscopy.

The procedure applied for SBA-15+AMETAM+GA was similar to that described above; 0.200 g of SBA-15 was suspended in toluene and an excess of AMETAM was added. The mixture was refluxed for 3 h and then stirred overnight at room temperature, filtered off, washed with toluene and dried. The amount of 0.274 g of SBA-15+AMETAM was obtained. A portion of 0.270 g of SBA-15+AMETAM was suspended in THF and DIPEA, followed by tri-O-acetylgalloyl chloride addition in a small excess with respect to the stoichiometric amount (that is 2 chloride molecules per each AMETAM group on the surface). The mixture was stirred at room temperature overnight and filtered off. The solid was washed carefully with THF, methanol and water and dried. The amount of 0.322 g of SBA-15+AMETAM+GA as a white solid was obtained.

The immobilization through PEI was carried out by a different method. A portion of 0.254 g of SBA-15 was suspended in toluene and 0.425 g of CPTMS in toluene was added. The mixture was refluxed for 5 h, stirred overnight at room temperature and filtered off. The solid was washed with toluene and dried. The amount of 0.267 g of SBA-15+CPTMS was obtained as a white solid. In the second step, 0.260 g of SBA-15+CPTMS was suspended in methanol and 0.565 g of PEI (50% water solution) along with small excess of DIPEA was added, the reagents were refluxed for 5 h, stirred overnight at room temperature and filtered off. The solid was washed with water and methanol and dried. The amount of 0.300 g of SBA-15+CPTMS+PEI as a white solid was obtained. Finally, 0.291 g of SBA-15+CPTMS+PEI was suspended in THF and tri-O-acetylgalloyl chloride along with DIPEA (in a small excess with respect to the chloride) were added. The mixture was stirred overnight at room temperature and filtered off. The solid was washed with water and methanol, then dried. The amount of 0.369 g of SBA-15+CPTMS+PEI+GA as a pale yellow solid was obtained.

**Fig 2:**
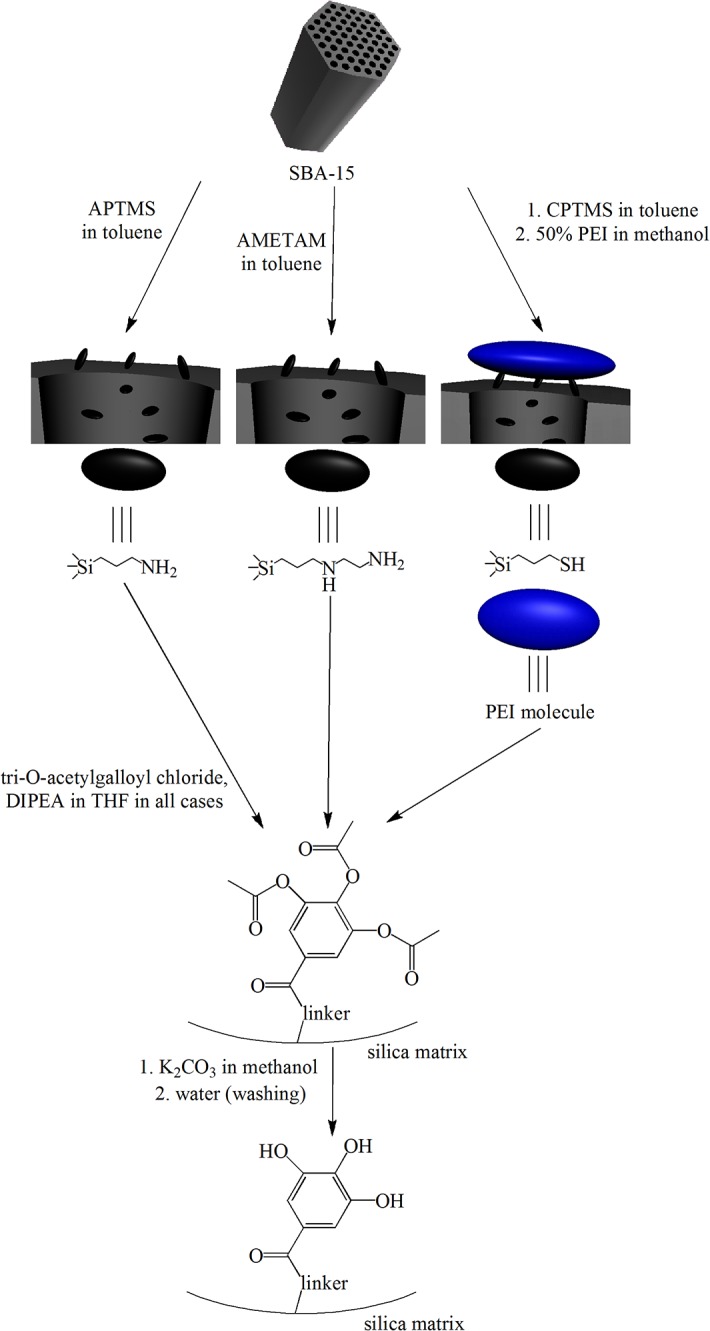
Schematic representation of the procedure used for the synthesis of gallic acid modified SBA-15 mesoporous silica.

In all procedures, the last step was the deprotection of acetyl groups. The procedure was adapted from Corey et al. [25] and carried out as follows: the modified silica sample was suspended in a saturated methanolic solution of K_2_CO_3_ and stirred at room temperature for 15 min (**Fig 2**). All final products changed colour during this step, starting with white, through pale pink ending with light brown. The solids were then filtered off, washed carefully with methanol and water, and dried. The presence of free phenol–OH groups could be quickly confirmed by suspending particles in a Fe^3+^ solution (which is slightly acidic). Deprotected products immediately formed dark violet complexes with Fe^3+^ ions, while the silica with acetyl-blocked phenol groups became violet after at least 5–10 min.

All systems obtained were characterized using elemental analysis and IR spectroscopy. Modified silica samples were also tested to check for uncontrolled gallic acid detachment. It was carried out by suspending samples of 20 mg of the composite materialsin water at different pH values (buffer solutions with integer pH values between 2 and 8) and stirring for 24 h. Then the suspensions were filtered and the filtrates' absorbances were measured on an UV-VIS spectrophotometer (Agilent 8453). No detachment was observed.

### Evaluation of the cytotoxic activity of the systems obtained

Human cancer cells HeLa (cervical cancer cell line) and KB (*carcinoma nasopharynx*) were cultured in RPMI 1640 medium. Each medium was supplemented with 10% fetal bovine serum, 1% L-glutamine and 1% penicillin/streptomycin solution. The cell lines were kept in the incubator at 37°C. The optimal plating density of cell lines was determined to be 5 x 10^4^. All the cell lines were obtained from The European Collection of Cell Cultures (ECACC) supplied by Sigma-Aldrich (catalogue numbers: HeLa cell line – 93021013, KB cell line – 94050408).

The protein-staining sulforhodamine B (SRB, Sigma-Aldrich) microculture colorimetric assay, developed by the National Cancer Institute (USA) for in vitro antitumor screening was used in this study, to estimate the cell number by providing a sensitive index of total cellular protein content, which is in a linear relationship to the cell density [26]. The monolayer cell culture was trypsinized and the cell count was adjusted to 5 x 10^4^ cells. In each well of the 96 well microtiter plate, 0.1 mL of the diluted cell suspension (approximately 10,000 cells) was placed. After 24 hours, when a partial monolayer was formed, the supernatant was washed out and 100 μL of six different silica suspension concentrations were added to the cells in microtiter plates. The tested silicas were suspended in DMSO (20 μM) and the content of DMSO did not exceed 0.1% as this concentration was found to be nontoxic to the cell lines. The cells were exposed to silicas for 72 h. After that, 25 μL of 50% trichloroacetic acid were added to the wells and the plates were incubated for 1 hour at 4°C. The plates were then washed out with distilled water to remove traces of medium and next dried by air. The air-dried plates were stained with 100 μL SRB and kept for 30 minutes at room temperature. The unbound dye was removed by rapidly washing with 1% acetic acid and then air dried overnight. The optical density was read at 490 nm. All cytotoxicity experiments were performed three times. Cell survival was measured as the percentage absorbance compared to the control (non-treated cells). Zidovudine (Sigma-Aldrich) was used as the internal standard.

## Results and Discussion

### Characterization of synthesized compounds

Melting point of acetylated gallic acid was found at 168–171°C, which is in agreement with literature data [27]. IR characterization (full spectrum available in the supplementary material) of acetylated gallic acid confirmed the presence of essential moieties. Comparatively low intensities observed in the range between 3100 and 2500 cm^-1^ suggest complete substitution of phenol–OH groups with acetyl ones. The signal at 1700 cm^1^ relates to the untouched carboxyl group (C = O stretching) and those at 1790 and 1770 cm^-1^ come from acetoxy groups (C = O stretching).

ESI MS analysis (full spectra available in the supplementary material) also unambiguously proves the presence of the desired product. ES- part represents loss of H^+^, carboxyl group and consecutive detachment of acetyl groups. ES+ part shows signals related to product’s complexes with sodium and potassium ions.

### Characterization of modified silica obtained

IR spectra (full spectra available in the supplementary material) of all obtained solids confirmed the presence of secondary amide bonds in the samples. The most characteristic signals, related to the C = O stretching can be found at 1640 cm^-1^, 1630 cm^-1^ and 1610 cm^-1^ for SBA-15+AMETAM+GA, SBA-15+APTMS+GA and SBA-15+CPTMS+PEI+GA, respectively. The differences between all these samples are a result of the formation of different types of amides that is to say secondary and tertiary ones. SBA-15+APTMS+GA may contain only secondary amide bonding, SBA-15+AMETAM+GA, secondary (mostly) and tertiary and SBA-15+CPTMS+PEI+GA—tertiary with a small addition of secondary one. The positions of signals from the stretching vibrations of the C = O group from SBA-15+APTMS+GA and SBA-15+AMETAM+GA differ only by 10 cm^-1^ (or even less, because precise location of both peaks is problematic as they are not sharp enough), which is a negligible difference and the position of the signal from SBA-15+CPTMS+PEI+GA differs more, because of the fact explained above. Numerous examples found in literature confirm that for amides, a descending order occurs that is the wavenumbers of amide C = O stretching signals of primary, secondary and tertiary amides decrease in that order. The spectra of all samples showed a signal at 1500 cm^-1^, which corresponds to the N-H deformation band (for SBA-15+APTMS+GA this signal is only a distortion of a stronger one). There is also no signal from acetyl groups (near 1800 cm^-1^) which proves that the deprotection process has occurred. Other signals, which can normally be assigned in the spectra of pure compounds, are lost because of the abundance of mesoporous silica or are not decisive ones.

As in SBA-15+APTMS+GA and SBA-15+AMETAM+GA samples the only sources of nitrogen were APTMS and AMETAM and there were two sources of carbon (nitrogen-containing linkers and the attached gallic acid), the elemental analyses allowed the calculation showing that SBA-15+APTMS+GA (10.11% C, 1.709% N and 2.275% H in total) contained 0.51 mmol of gallic acid per gram of the modified silica, which gave about 40% of nitrogen atoms covered with gallic acid. The same calculations conducted for the SBA-15+AMETAM+GA (14.31% C, 3.806% N and 3.380% H) led to 0.64 mmol of gallic acid per gram of the modified silica and 23% of nitrogen atoms coverage. The calculations for the SBA-15+CPTMS+PEI+GA (13.56% C, 3.313% N and 3.073% H) required additional analyses (three carbon-containing sources) and finally led to the result of 0.59 mmol of gallic acid per gram of the modified silica and averaged 25% of nitrogen atoms coverage. The coverage differs in all samples because of the spatial issues (acetylated GA occupies a lot of space) and nitrogen atoms order. APTMS contains only primary amino groups—easily accessible and reactive. AMETAM introduced more than twice as many nitrogen atoms as APTMS, equally primary and secondary ones. Probably most of the primary and some part of secondary nitrogen atoms reacted, but the total coverage (due to high total amount of nitrogen) is lower than that of the APTMS. PEI introduced less nitrogen (as primary, secondary and tertiary atoms) and allowed to anchor less GA than AMETAM, mainly due to its steric properties. Low accessibility of nitrogen atoms resulted in low coverage.

### Evaluation of cytotoxic activity

**Fig 3:**
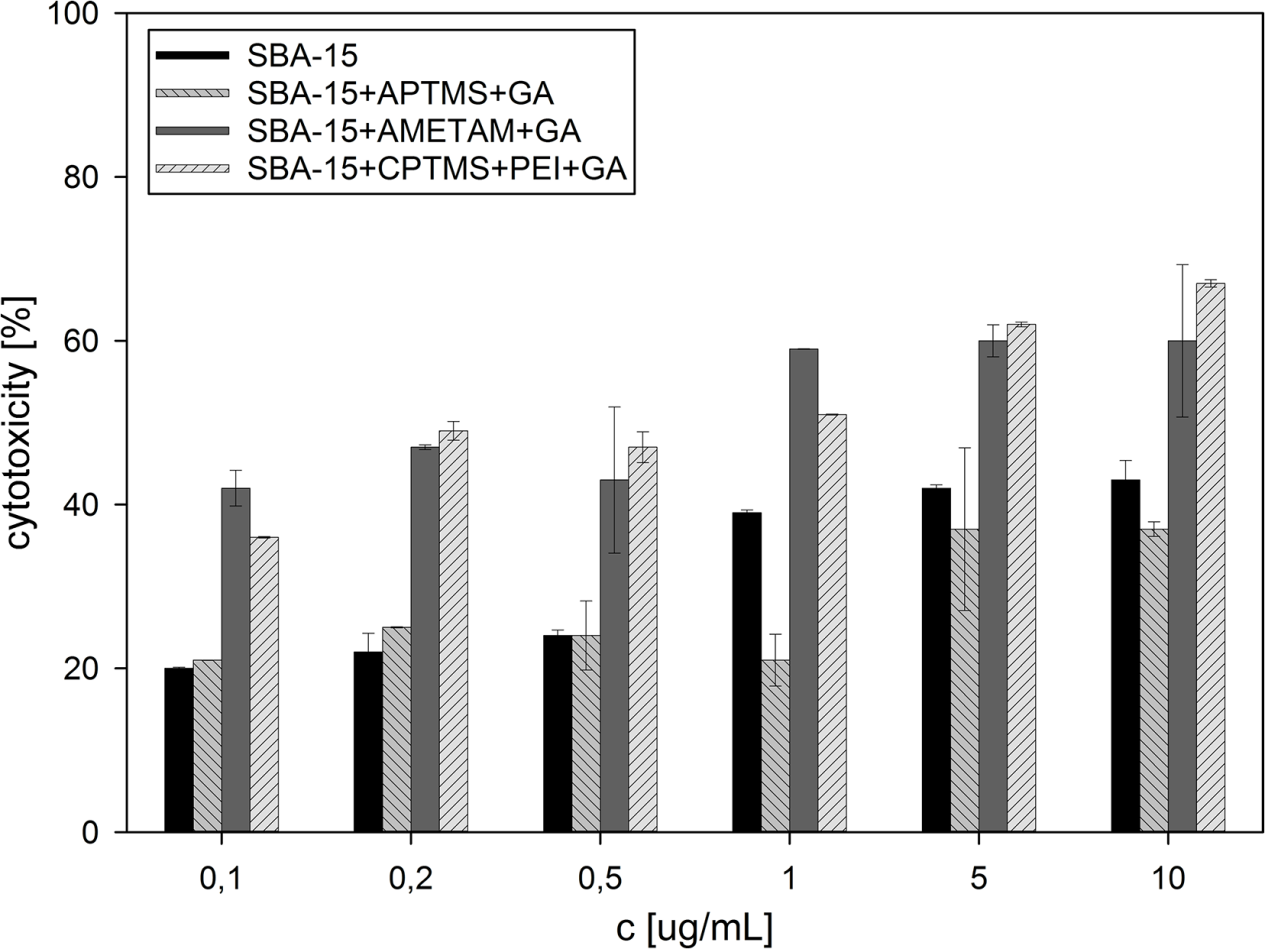
Cytotoxic activity against HeLa cell line, calculated for all samples tested.

**Fig 4:**
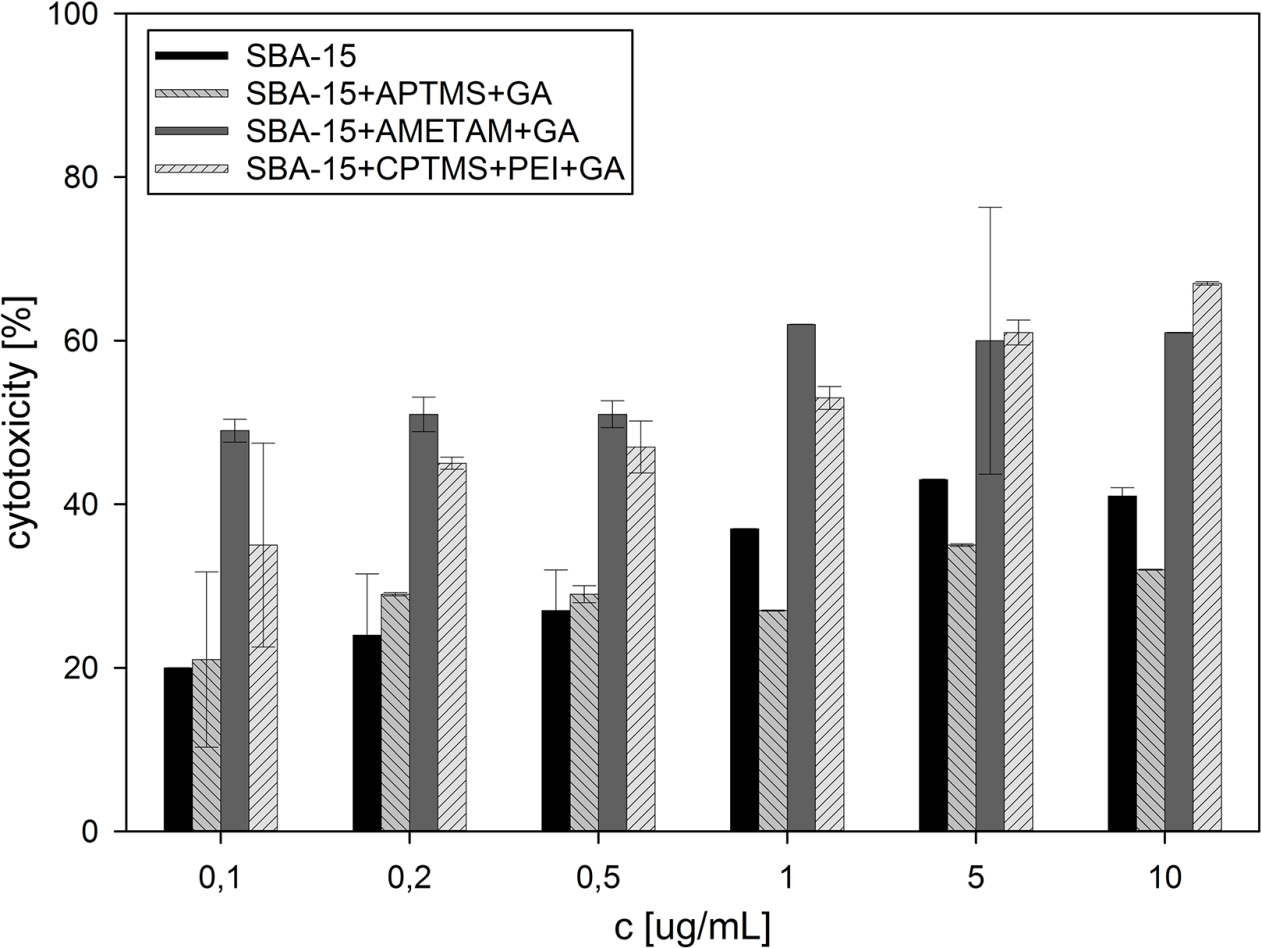
Cytotoxic activity against KB cell line, calculated for all samples tested.

All prepared and analyzed solids showed concentration-dependent cytotoxic activity against HeLa and KB cell lines (**Fig 3 and Fig 4**) which are used very often in such studies.

The sample modified with APTMS and gallic acid was not as active as the unmodified silica, though it contained 0.51 mmol of gallic acid per gram of the solid, which is about 8% in weight. That minor difference can be explained by the fact that the surface coverage drastically reduced the accessibility of acidic silanol groups (which are the active agents in bare silica particles) and, in exchange, introduced a combination of primary amino groups (potentially increasing the activity and enhancing the uptake) and phenolic hydroxyl groups (much less numerous in comparison to silanol ones), which proved to be less active. Two other samples, with gallic acid anchored to the surface through AMETAM and a combination of CPTMS and PEI, showed much higher activity, equalling or even surpassing that of pure Zidovudine (IC_50_ = 3.12 μgmL^-1^ for KB and 2.28 μgmL^-1^ for HeLa cell line). The activity of SBA-15+AMETAM+GA increased slowly at concentrations up to 1 μg mL^-1^ and then remained at the same level, even at the concentration ten times higher. The activity of SBA-15+CPTMS+PEI+GA increased steadily as the concentration rose up to 10 μg mL^-1^, and surpassed that of SBA-15+AMETAM+GA between 1 and 5 μg mL^-1^. This might be a result of differences in the non-specific interactions with cell membrane that influenced the cellular uptake. In these samples the amount of gallic acid on the surface was noticeably higher and the percentage of nitrogen atoms covered with gallic acid was lower than in SBA-15+APTMS+GA. These two features can be related to the observed increase in activity (in standard organic compounds, introduction of the amino group generally increases their biological activity [28,29,30]) and, what is also worth noticing, free amino groups in SBA-15+AMETAM+GA and SBA-15+CPTMS+PEI+GA are mainly secondary and tertiary ones. High deviations of the results obtained for some of the samples are a result of sedimentation in silica suspensions (statistical data available in the supplementary material).

## Conclusions

Gallic acid was successfully introduced onto the SBA-15 mesoporous silica surface using different linkers. Cytotoxic activity against HeLa and KB cell lines of all solids obtained has been evaluated. The values of cell growth inhibition obtained for the samples are relatively high and equal up to 67% for HeLa and KB tumor cells at low silica suspension concentration. The results of this study can be a basis for further attempts at covalent conjugation of gallic acid and other polyphenols to the silica surface.

## Supporting Information

S1 Spectrum Full FT-IR spectrum of acetylated gallic acid. (TIF)

S2 Spectrum ESI MS negative and positive spectra of acetylated gallic acid. (TIF)

S3 Spectrum IR spectra of SBA-15 mesoporous silica modified with gallic acid. (TIF)

S1 Table Statistical data from the cytotoxicity assay. (DOCX)
