# Supplementary figures and images for: SBA-15 Mesoporous Silica Modified with Gallic Acid and Evaluation of Its Cytotoxic Activity
Source: PLoS One. 2015 Jul 7;10(7):e0132541. doi: 10.1371/journal.pone.0132541 (PMC4495030; doi:10.1371/journal.pone.0132541)

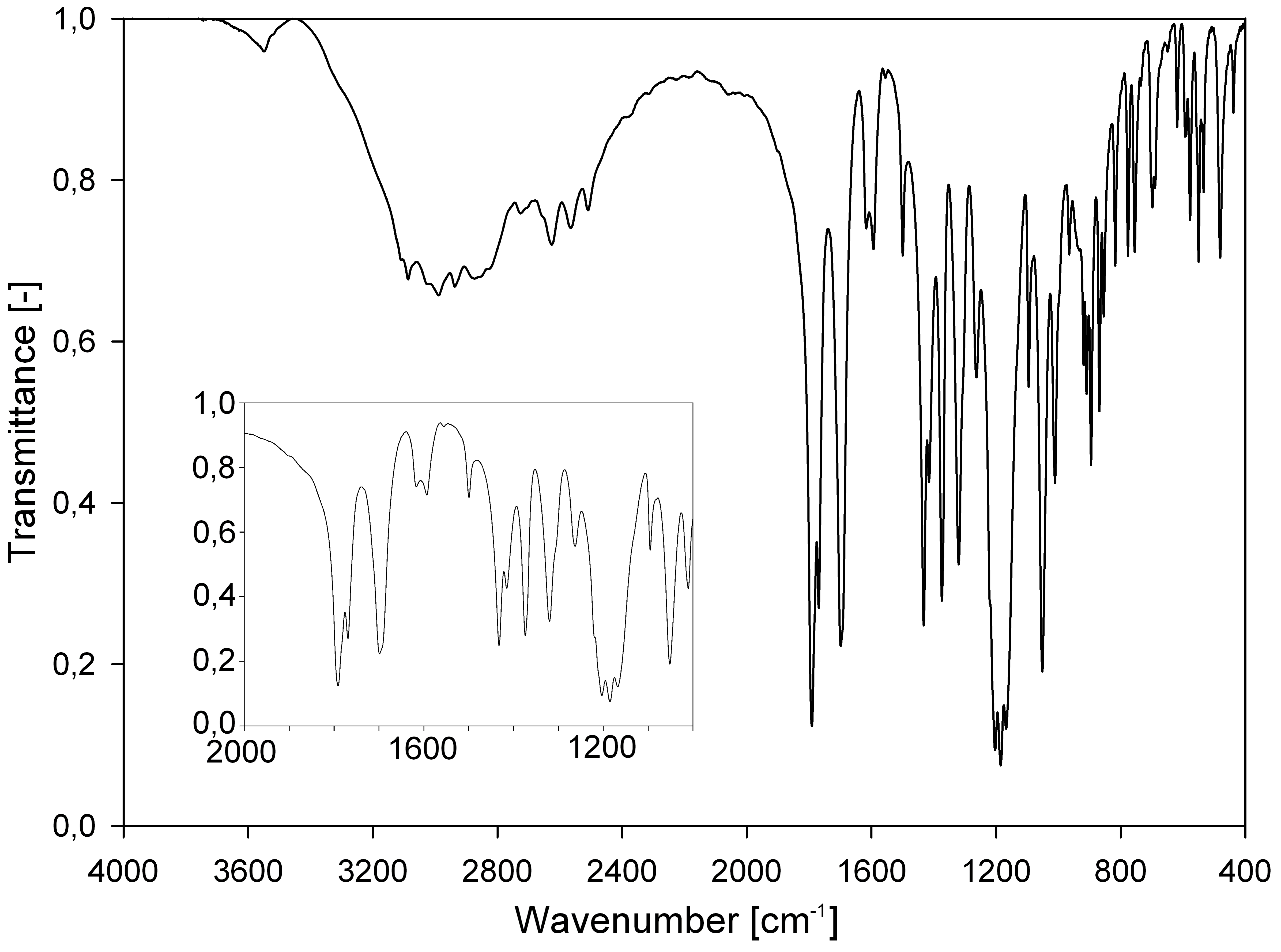

Supplement: S1 Spectrum — (TIF) [file pone.0132541.s001.tif]

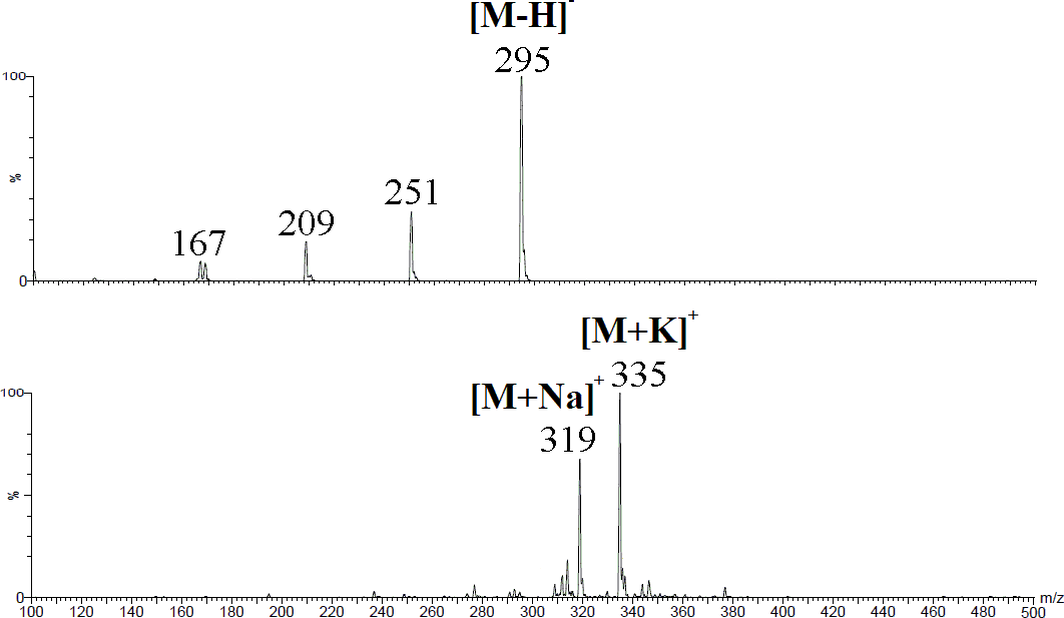

Supplement: S2 Spectrum — (TIF) [file pone.0132541.s002.tif]

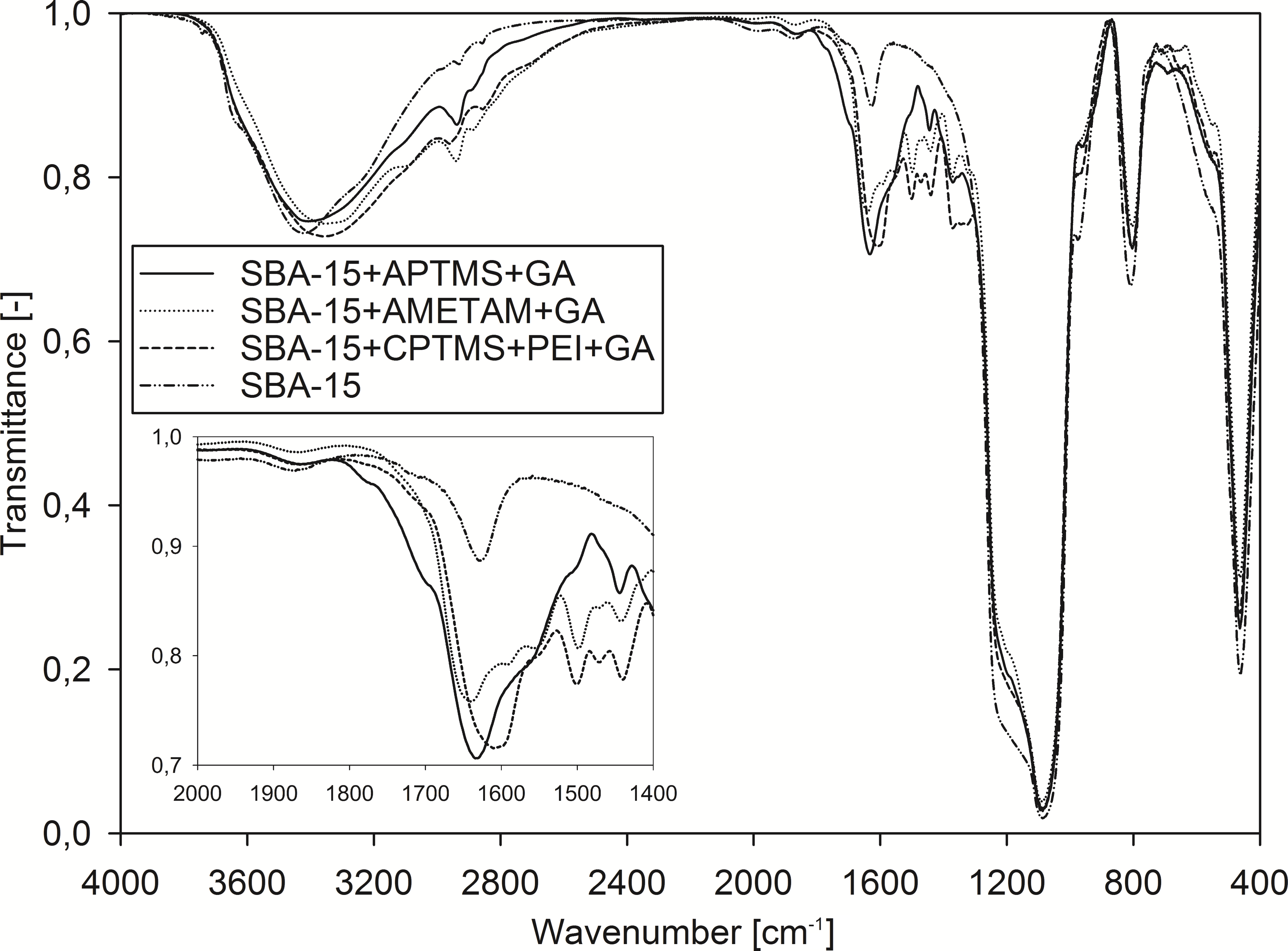

Supplement: S3 Spectrum — (TIF) [file pone.0132541.s003.tif]
