# Supplementary material for: SBA-15 Mesoporous Silica Modified with Gallic Acid and Evaluation of Its Cytotoxic Activity
Source: PLoS One. 2015 Jul 7;10(7):e0132541. doi: 10.1371/journal.pone.0132541 (PMC4495030; doi:10.1371/journal.pone.0132541)
Supplement: S1 Table — (DOCX) [file pone.0132541.s004.docx]

| Silica suspension concentration [µg/mL] | Cell line | Cells growth inhibition [%] with standard deviation (in brackets) obtained for: | | |
| --- | --- | --- | --- | --- |
|  |  | SBA-15+ APTMS+GA | SBA-15+ AMETAM+GA | SBA-15+ CPTMS+PEI+GA |
| 0.1 | HeLa | 21 (0.01) | 42 (2.18) | 36 (0.09) |
|  | KB | 21 (10.71) | 49 (1.41) | 35 (12.47) |
| 0.2 | HeLa | 25 (0.06) | 47 (0.29) | 49 (1.16) |
|  | KB | 29 (0.19) | 51 (2.11) | 45 (0.74) |
| 0.5 | HeLa | 24 (4.21) | 43 (8.92) | 47 (1.88) |
|  | KB | 29 (1.04) | 51 (1.64) | 47 (3.18) |
| 1.0 | HeLa | 21 (3.17) | 59 (0.03) | 51 (0.06) |
|  | KB | 27 (0.05) | 62 (0.01) | 53 (1.40) |
| 5.0 | HeLa | 37 (9.93) | 60 (1.95) | 62 (0.29) |
|  | KB | 35 (0.14) | 60 (16.32) | 61 (1.51) |
| 10.0 | HeLa | 37 (0.89) | 60 (9.30) | 67 (0.45) |
|  | KB | 32 (0.07) | 61 (0.03) | 67 (0.21) |
